# The Usefulness of Mayo End-stage Liver Disease (MELD) and MELD-Sodium (MELD-Na) Scores for Predicting Mortality in Cirrhotic Patients With Spontaneous Bacterial Peritonitis

**DOI:** 10.7759/cureus.38343

**Published:** 2023-04-30

**Authors:** Catherine Coxeter-Smith, Ali Al-Adhami, Laith Alrubaiy

**Affiliations:** 1 Medical School, Imperial College London, London, GBR; 2 Gastroenterology and Hepatology, St Mark's Hospital, London, GBR; 3 Gastroenterology, St Mark's Hospital, London, GBR

**Keywords:** 90-day mortality, model for end-stage liver disease sodium, model for end-stage liver disease, cirrhosis, spontaneous bacterial peritonitis

## Abstract

Background: Spontaneous bacterial peritonitis (SBP) is a common infection in patients with cirrhosis and ascites. Currently, the accuracy of the model for end-stage liver disease (MELD) and MELD-sodium (MELD-Na) as prognostic scores in this cohort is unclear. This study aimed to evaluate and compare the accuracy of MELD and MELD-Na for predicting 90-day mortality and determine whether the mortality risk estimates they provide accurately reflect the poor prognosis of patients with SBP

Methods: Patients with cirrhosis and SBP were retrospectively identified from ascitic fluid samples sent for microscopy, culture and sensitivity analysis (1/1/18-31/12/20) and a previous audit. MELD and MELD-Na scores at diagnosis were calculated and associations with 90-day mortality were assessed using univariate analysis. Receiver operator characteristic curves were compared, and standardised mortality ratios (SMRs) were calculated by comparing the number of deaths observed to the number predicted by MELD and MELD-Na.

Results: Of the 567 patients identified, 15 patients with cirrhosis and SBP were included. The 90-day mortality rate was 66.7% (10/15). Only concurrent hyponatremia (<135mmol/L) was associated with mortality (6/10 non-survivors vs 0/5 survivors, p=0.04). The difference in MELD and MELD-Na’s C-statistic was not significant: 0.66 (95% Cl:0.35-0.98) vs 0.74 (95% Cl:0.47-1.0) respectively (p=0.72). Patients with a MELD-Na >18.5 had significantly higher 90-day mortality than patients with MELD-Na ≤18.5 (88.9% (8/9) vs. 33.3% (2/6), p=0.05). The SMR (95% Cl) for each MELD decile evaluated was 33.3 (0-79.5), 11.1 (0.2-22.0) and 3.4 (0-7.0) for scores ≤9,10-19 and 20-29 respectively. For each MELD-Na tertile, these were: 25 (0-59.6), 5.2 (0.1-10.3) and 2.7 (0.1-8.1) for scores <17,17-26, ≥27 respectively.

Conclusion: In a small cohort of patients with cirrhosis and SBP, the MELD’s accuracy in predicting 90-day mortality was limited. MELD-Na’s accuracy was higher but not significantly. Both scores consistently underestimated participants’ mortality, therefore future studies could evaluate the accuracy of alternative prognostic scores in this patient group.

## Introduction

Spontaneous bacterial peritonitis (SBP) is defined as a bacterial infection of the ascitic fluid that occurs in the absence of any surgically treatable intra-abdominal sources of infection [1]. SBP is common in patients with cirrhosis and ascites [2,3] as a result of increased bacterial translocation from the gastrointestinal tract to mesenteric lymph nodes and other extra-intestinal sites [4]. Due to impaired local [5] and systemic [6] immune defences, these microorganisms, most commonly Gram-negative enteric bacteria [7] may not be killed effectively and therefore can colonise the ascitic fluid [4]. Presentation with SBP varies; some patients are asymptomatic and are diagnosed incidentally during large-volume paracentesis [8] whereas others present with severe sepsis and multiorgan failure [9,10]. Despite vast improvements in the early diagnosis and treatment of SBP [11], the short-term prognosis is poor; in-hospital and 90-day mortality is 36% and 45% respectively [12]. 

The model for end-stage liver disease (MELD) is a widely used prognostic score in patients with cirrhosis. Originally designed to predict short-term survival following elective transjugular intrahepatic portosystemic shunt (TIPS) placement [13], it subsequently showed excellent ability to predict 90-day mortality in patients on the liver transplant waiting list in the United States [14,15]. Therefore, in 2002, MELD replaced Child-Turcotte-Pugh as the basis for prioritising donor livers to patients on the waiting list with the poorest short-term prognosis [16]. MELD is calculated from three laboratory variables: serum creatinine, serum bilirubin and international normalised ratio (INR). Scores range from 6-40; higher scores indicate more severe liver disease and are associated with a higher 90-day mortality risk [14].

Outside of the context of donor liver allocation, MELD has also been validated as a prognostic score in multiple other cohorts: for example, to predict short-term mortality in patients with cirrhosis undergoing elective and emergency surgery [17] and in alcohol-related hepatitis [18]. Therefore, even in the United Kingdom (UK) where the UK model for end-stage liver disease (UKELD) is used to determine eligibility for liver transplant and donor liver allocation instead [19] MELD remains a commonly used prognostic score.

At present, few studies have formally evaluated the predictive accuracy of MELD in patients with cirrhosis and SBP and the results so far have been conflicting [10,20,21] both when multivariate analysis has been utilised to determine whether MELD is an independent predictor of mortality, and when the model’s discriminatory ability has been evaluated using receiver operator characteristic (ROC) curves. Identifying prognostic scores with high predictive accuracy in this cohort may allow patients to be risk-stratified to help identify, alongside clinical judgement, those which require closer monitoring or more intensive treatment. This may also be of use in clinical trials to help provide a clearer understanding of the effectiveness of new therapies in different risk groups.

Secondly, it is unclear whether the mortality risk estimates associated with each decile of the MELD score accurately represent the high short-term mortality of patients with SBP [22]. Previously, MELD has been shown to underestimate the mortality of patients with other cirrhosis-related complications such as hepatic encephalopathy [23] and persistent ascites [24]. Additionally, in the original MELD validation cohort, less than 1% of patients had SBP [14], however prospective studies have shown this infection to confer a significantly poorer 90-day outcome compared to non-infected patients [25]. Identifying prognostic scores which provide accurate mortality risk estimates in this cohort may aid clinicians in clearly communicating information on prognosis and disease trajectory to patients and their families. Importantly, frequent admissions to hospitals with decompensated cirrhosis such as with SBP are recognised as important events to prompt potential referral to palliative care [26,27]. Whilst this has been shown to improve patient quality of life and reduce symptom burden [28], lack of understanding of prognosis by patients has been identified as an important factor for the unwillingness of some to engage with these services [29].

A commonly utilised modification of MELD is the model for end-stage liver disease sodium (MELD-Na). Hyponatremia, which develops in patients with cirrhosis due to excess fluid retention disproportionate to that of sodium [30] has been widely associated with a poorer prognosis in this patient group [31,32]. The model’s inclusion of serum sodium concentration alongside the three original MELD components has shown a superior ability to predict 90-day mortality in patients on the liver transplant waiting lists in both the United States [33] and Europe [34]. Whilst hyponatremia appears to be especially common in patients with cirrhosis and infections such as SBP [31], MELD-Na’s prognostic accuracy has yet to be widely evaluated or compared to MELD alone in this cohort.

This retrospective study aims to evaluate and compare the predictive accuracy of MELD and MELD-Na for predicting 90-day mortality in patients with cirrhosis and SBP. In addition, by comparing the mortality of study participants to the mortality-risk estimates associated with each MELD [15] and MELD-Na [33] score, this study aims to assess whether these models may underestimate the 90-day mortality of this patient group.

## Materials and methods

Study design

This was a retrospective study conducted as part of a local quality improvement project at Northwick Park Hospital (approved in April 2021). No formal ethical approval was required, and no patient-identifiable information was collected. Patient management was not affected.

Patients

To identify eligible patients for this study, all consecutive ascitic fluid samples sent for microscopy, culture and sensitivity analysis at London Northwest University Healthcare NHS Trust (Northwick Park and Ealing Hospitals) between 1st January 2018 and 31st December 2020 were considered retrospectively. In addition, patients diagnosed with SBP between 1st May 2019 and 30th April 2020 at Imperial College Healthcare NHS Trust (St. Mary’s, Hammersmith and Charing Cross Hospitals) who had been identified prior to the start of the present study for audit purposes were considered for eligibility.

The inclusion criteria were adults (≥18 years of age) with cirrhosis and an ascitic fluid neutrophil count >250 cells/mm3 (culture-positive or negative) [9]. The presence of cirrhosis was noted from radiologists’ reports that accompanied imaging on or prior to the date of the diagnostic paracentesis which confirmed SBP. Patients were included irrespective of any history of SBP.

The exclusion criteria were any evidence of a surgically treatable intra-abdominal source of infection [9], active malignancy (hepatic and/or extrahepatic) and patients with human immunodeficiency virus (HIV). Patients who underwent a liver transplant before diagnostic paracentesis or in the following 90 days were also excluded.

For patients who experienced multiple admissions with SBP during the time periods included, only the first admission was included.

Data collection

Patients’ demographic and clinical information at presentation including age, sex, ethnicity and aetiology of cirrhosis was obtained from electronic clinical records. The presence of concurrent acute kidney injury (AKI), upper gastrointestinal haemorrhage and hepatic encephalopathy on presentation with SBP was noted from electronic patient notes and discharge summaries. Hyponatremia was defined as a serum sodium concentration of <135mmol/L33 and was noted using laboratory findings at the time of abdominal paracentesis.

The study outcome was all-cause mortality at 90 days from the date of diagnostic paracentesis which was also noted from electronic patient records.

MELD and MELD-Na scores

Prognostic scores were calculated from laboratory tests that occurred within the 24 hours directly preceding or following diagnostic paracentesis. Where multiple tests had been completed within this timeframe, the test performed closest in time to that of the paracentesis was used to calculate a patient’s score.

MELD scores were calculated using an online calculator [35] with the formula: (0.957 x In(Serum Creatinine) + 0.378 x In(Serum Bilirubin) + 1.120 x In(INR) + 0.643) x 10.

MELD-Na scores were calculated using an online calculator [36] with the formula: MELD - Serum Na - 0.025 x MELD x (140 - Na) + 140.

Statistical analysis

Continuous variables were assessed for normality using the Shapiro-Wilk test. Results are presented as means and standard deviations for continuous parametric data, medians and interquartile ranges for continuous non-parametric data and frequencies and percentages for categorical data. Univariate analysis was performed to assess for associations with 90-day mortality using unpaired, two-tailed t-test, Mann-Whitney U test and Fischer’s exact test for each data type respectively.

ROC curves for each model were plotted and the accuracy of MELD and MELD-Na to predict 90-day mortality was assessed using concordance (c)-statistics (equivalent to the area under the ROC curve). C-statistic values range from 0-1; higher values indicate that a model can rank patients according to their outcomes more accurately. A c-statistic greater than 0.7 suggests a model is clinically useful; a score between 0.8-0.9 indicates excellent discriminatory ability [15]. C-statistics were compared using DeLong’s test [37].

The specificity and sensitivity of each MELD and MELD-Na cut-off were assessed visually for that with the highest values to determine if a specific value of MELD and MELD-Na could act as a threshold for potential risk-stratification. Kaplan-Meier survival curves for patients above and below the cut-off chosen were compared using the log-rank test.

To evaluate whether MELD and MELD-Na underestimated the mortality of study participants, standardised mortality ratios (SMRs) were calculated. Patients were stratified by MELD score into deciles (≤9, 10-19, 20-29, 30-39, ≥40) and by MELD-Na score into tertiles (<17, 17-26 and ≥27). In each grouping, the number of deaths expected according to MELD and MELD-Na was calculated using the 90-day mortality rates published by Wiesner et al. [15] and Kim et al. [33] respectively as these represent the widely quoted mortality risk estimates associated with each score [35,36]. The number of expected deaths was then compared to the number observed in the present study (SMR=observed/predicted number of deaths). An SMR greater than one indicates that mortality has been underestimated by a prognostic score [38].

All analyses were conducted using GraphPad Prism v9.1 (GraphPad Software, San Diego, CA) except for the calculation of SMRs where Microsoft Excel (v16.49) was used. For all tests, a p-value of ≤0.05 was considered significant except for SMRs, where a 95% confidence interval that did not cross one was considered significant.

## Results

**Figure 1:**
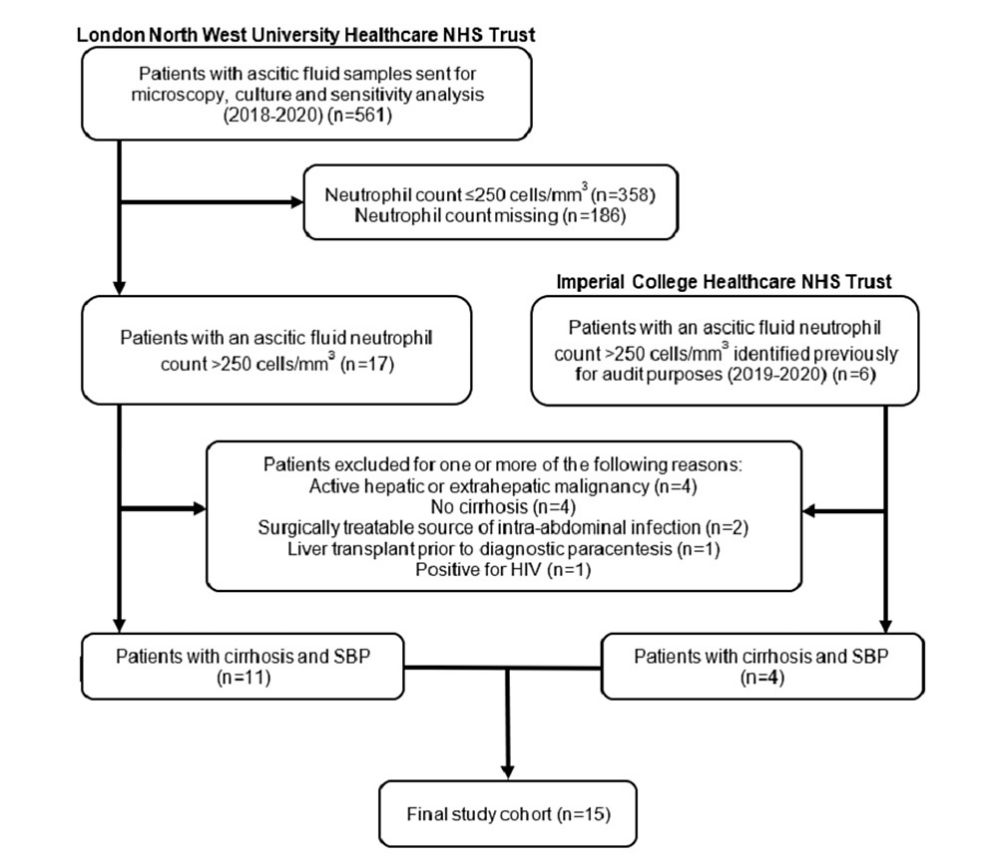
Flowchart showing the screening of eligible patients and application of the study’s inclusion and exclusion criteria at both NHS trusts to produce the final study cohort (n=15). NHS: National Healthcare Service; HIV: human immunodeficiency virus; SBP: spontaneous bacterial peritonitis.

Across the two NHS trusts, 567 patients in total were considered for eligibility. As shown in Figure 1, after the inclusion and exclusion criteria were applied, 15 patients with cirrhosis and SBP were identified. 

Patients and outcome

The demographic and clinical characteristics of the study cohort and laboratory findings at diagnosis with SBP are shown in Table 1. Overall, the mean age was 58.4 ±10.3 years. Around 12 out of 15 patients were male and almost all patients were either Asian or Asian British (7/15) or White (6/15).

**Table 1:** Demographic, clinical and laboratory information for the overall study cohort and factors associated with 90-day mortality. ^a ^Continuous, parametric data expressed as mean ±SD. ^b ^Continuous, non-parametric data expressed as median (IQR). * p≤0.05 between non-survivors and survivors Data compared between 90-day non-survivors and survivors using unpaired, two-tailed t-test (continuous, parametric data), Mann Whitney U test (continuous, non-parametric data) and Fisher’s exact test (categorical data). All statistical tests were two-tailed and p≤0.05 was considered significant. ALD: alcoholic liver disease; NAFLD: non-alcoholic fatty liver disease; PBC: primary biliary cholangitis; SBP: spontaneous bacterial peritonitis; GI: gastrointestinal; MELD: model for end-stage liver disease; MELD-Na: model for end-stage liver disease sodium; INR: international normalised ratio; WBC: white blood cell; CRP: c-reactive protein; SD: standard deviation; IQR: interquartile range.

		Outcome at 90-days		
	Overall (n=15)	Non-survivors (n=10)	Survivors (n=5)	p-value
Age^a^ – yr	58.4 ±10.3	60.3 ±10.4	54.6 ±10.1	0.33
Male sex – no. (%)	12 (80)	8 (80)	4 (80)	>0.99
Ethnicity – no. (%)				
Asian or Asian British	7 (46.7)	4 (40)	3 (60)	0.61
White	6 (40)	4 (40)	1 (20)	
Other	2 (13.3)	1 (10)	1 (20)	
Aetiology of cirrhosis – no. (%)				
ALD	10 (66.7)	6 (60)	4 (80)	0.60
Hepatitis C	2 (13.3)	2 (20)	0	
NAFLD + ALD	2 (13.3)	2 (20)	0	
PBC	1 (6.7)	0	1 (20)	
Culture-positive SBP – no. (%)	5 (33.3)	4 (40)	1 (20)	0.60
Escherichia coli	4 (26.7)	3 (30)	1 (20)	
Klebsiella pneumoniae	1 (6.7)	1 (10)	0	
Clinical features on presentation – no. (%)				
Acute kidney injury	6 (40)	5 (50)	1 (20)	0.28
Hyponatremia (<135mmol/L)	6 (40)	6 (60)	0	0.04*
Hepatic encephalopathy	5 (33.3)	4 (40)	1 (20)	0.60
Upper GI haemorrhage	1 (6.7)	0	1 (20)	0.33
Prognostic scores				
MELD^a^	18.9 ±7.3	17.8 ±6.5	14.4 ±6.5	0.36
MELD-Na^a^	20.8 ±6.9	21.8 ±6.8	15.4 ±6.4	0.10
Laboratory findings				
Creatinine^b^ – µmol/L	95.0 (71.3-132.3)	118.0 (66.8-300.8)	90.0 (65.5-124.0)	0.31
Bilirubin^b^ – µmol/L	29.0 (12.5-88.0)	30.5 (7.8-87.5)	29.0 (17.0-47.0)	0.70
INR^a^	1.5 ±0.5	1.4 ±0.4	1.5 ±0.3	0.41
Sodium^a^ – mmol/L	136.3 ±7.8	132.7 ±7.6	139.2 ±1.8	0.09
WBC^a^ – x10^9^/L	11.8 ±8.0	14.7 ±7.9	6.2 ±4.8	0.07
CRP^b^ – mg/L	56.7 (32.2-77.5)	67.7 (18.1-87.3)	47.3 (35.3-66.2)	0.95
Serum albumin^a^ – g/L	27.7 ±7.0	26.4 ±7.1	31.8 ±5.9	0.18

Of the 15 patients included, alcoholic liver disease (ALD) was the most common cause of underlying cirrhosis, affecting 10 patients. Hepatitis C and the combination of non-alcoholic fatty liver disease (NAFLD) and ALD were each responsible for cirrhosis in two patients. In the remaining patient, primary biliary cholangitis (PBC) was the cause of cirrhosis.

Alongside SBP, five patients also presented with an AKI and five with hyponatremia. One patient presented with both additional features concurrently. Five out of the 15 patients had hepatic encephalopathy at diagnosis, and one patient also presented with an upper gastrointestinal haemorrhage. Of the five patients with culture-positive SBP, the organism was identified as Escherichia coli in four cases and as Klebsiella pneumoniae in the remaining patient.

The mean MELD score at the time of diagnosis was 18.9 ±7.3 and the mean MELD-Na score was 20.8 ±6.9. At 90 days, 10 patients out of 15 patients included had died (66.7%). The median survival time was 45 days.

Factors associated with 90-day mortality

As shown in Table 1, only the presence of hyponatremia at diagnosis with associated with 90-day mortality: 60% (6/10) of patients who died were hyponatraemic compared to none of the five patients who survived (p=0.04). 

Other variables that were close to reaching statistical significance included MELD-Na score and white blood cell (WBC) count, both of which were higher in the non-survivors; 21.8 ±6.8 compared to 15.4 ±6.4 (p=0.10) and 14.7 ±7.9 x109/L compared to 6.2 ±4.8 x109/L (p=0.07) respectively. Serum sodium and albumin were both lower in non-survivors: 132.7 ±7.6 mmol/L compared to 139.2 ±1.8 mmol/L (p=0.09) and 26.4 ±7.1 g/L compared to 31.8 ±5.9 g/L (p=0.18) respectively.

MELD score was higher in patients who died compared to patients who survived; 17.8 ±6.5 compared to 14.4 ±6.5 (p=0.36). Of the three components of MELD, creatinine was also higher in non-survivors; 118.0 (66.8-300.8) µmol/L compared to 90.0 (65.5-124.0) µmol/L (p=0.31). In contrast, bilirubin and INR were almost identical between the two groups: bilirubin was 30.5 (7.8-87.5) µmol/L in non-survivors compared to 29.0 (17.0-47.0) µmol/L in survivors (p=0.70), whilst INR was 1.4 ±0.4 compared to 1.5 ±0.3 in non-survivors and survivors respectively (p=0.41).

Predictive accuracy of MELD and MELD-Na

**Figure 2:**
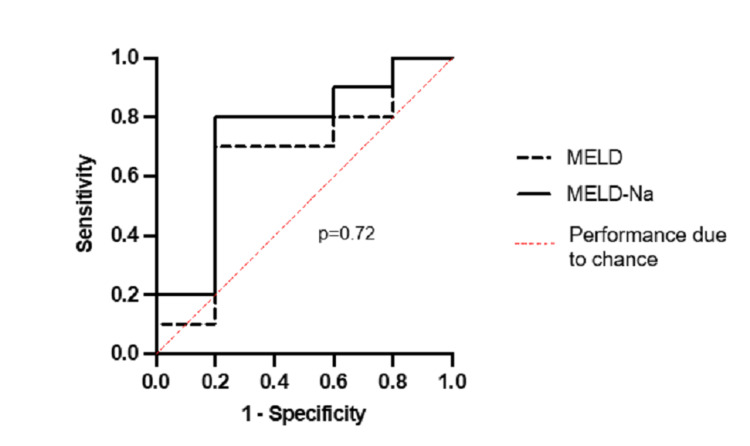
Comparison of ROC curves for MELD (-- dashed line, c-statistic: 0.66, 95% Cl:0.35-0.98) and MELD-Na (– black line, c-statistic: 0.74, 95% Cl:0.47-1.0) for predicting 90-day mortality in patients with cirrhosis and SBP. The difference in ROC curves was not significant (p=0.72). C-statistics were compared using DeLong’s test. p ≤0.05 was considered significant. MELD: model for end-stage liver disease; MELD-Na: model for end-stage liver disease sodium; ROC: receiver operator characteristic; c-statistic: concordance-statistic; SBP: spontaneous bacterial peritonitis; Cl: confidence interval

Figure 2 shows the ROC curves for MELD and MELD-Na predicting 90-day mortality. The c-statistic for MELD was 0.66 (95% Cl: 0.35-0.98) compared to 0.74 (95% Cl: 0.47-1.0) for MELD-Na. This difference was not significant (p=0.72).

**Figure 3:**
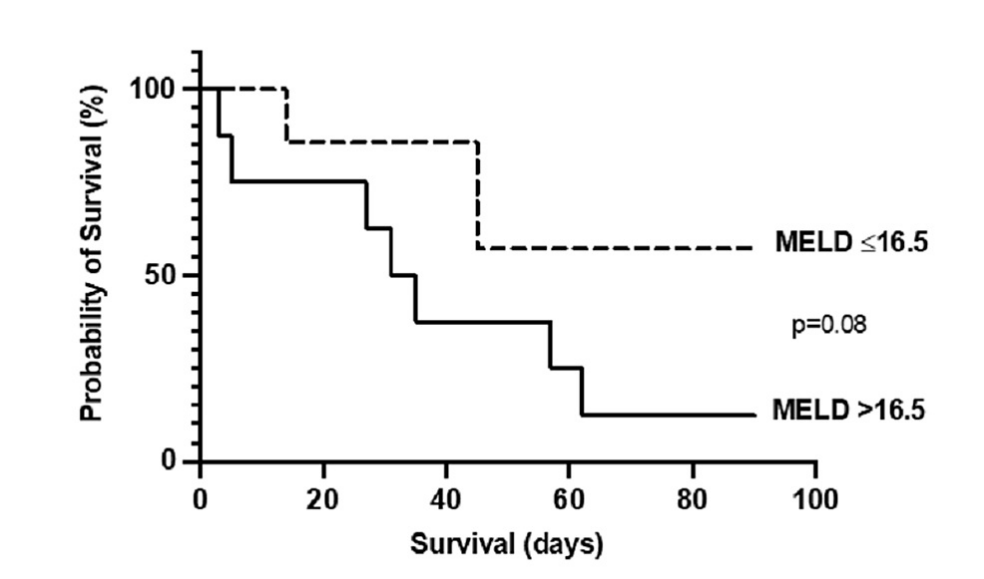
Comparison of 90-day mortality in patients with SBP stratified by MELD >16.5 Kaplan-Meier curves were compared using a log-rank test. p ≤0.05 was considered significant. MELD: model for end-stage liver disease; SBP: spontaneous bacterial peritonitis.

The MELD score with the highest sensitivity and specificity to predict 90-day mortality was a cut-off of >16.5, with values of 0.7 and 0.8 respectively. Figure 3 shows the difference in 90-day mortality when patients were stratified using this threshold. Patients with a score >16.5 had a 90-day mortality rate of 87.5% (7/8) compared to 42.9% (3/7) in those with a score of ≤16.5. This difference in mortality was not significant (p=0.08).

**Figure 4:**
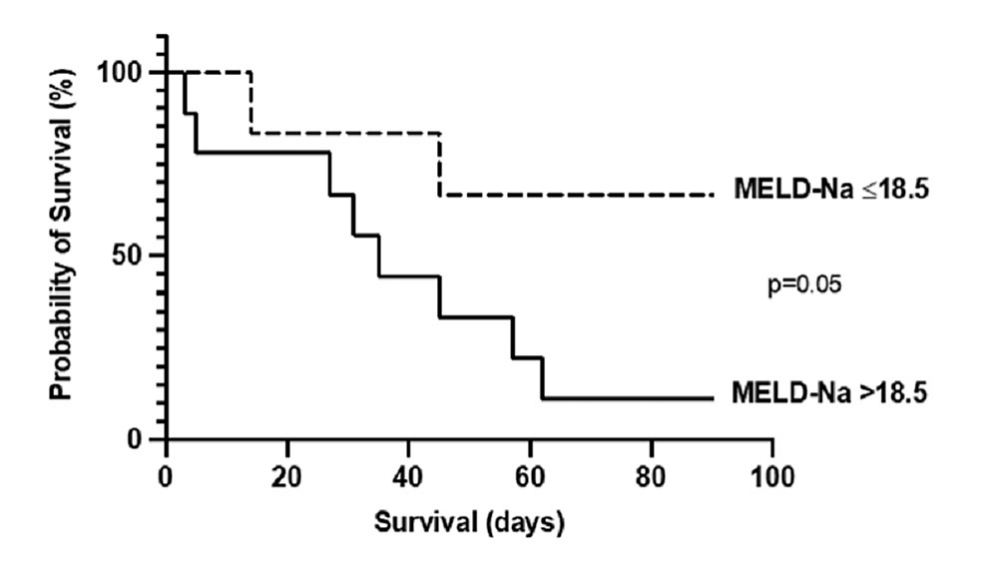
Comparison of 90-day mortality in patients with SBP stratified by MELD-Na >18.5 Kaplan-Meier curves were compared using a log-rank test. p ≤0.05 was considered significant. MELD-Na: model for end-stage liver disease sodium; SBP: spontaneous bacterial peritonitis.

The optimum cut-off for MELD-Na to predict 90-day mortality was a score of >18.5; sensitivity and specificity were both 0.8. As shown in Figure 4, the 90-day mortality rate of patients with a MELD-Na >18.5 was 88.9% (8/9) compared to 33.3% (2/6) in those scoring ≤18.5 (p=0.05).

Accuracy of MELD and MELD-Na’s mortality risk estimates

For each decile of the MELD score evaluated, the SMR was greater than one as the number of deaths observed was higher than the number predicted by MELD (Table 2). As the decile of the MELD score increased, the magnitude of this underestimation decreased: patients with a MELD score ≤9 had an SMR of 33 (95% Cl: 0-79.5) suggesting 90-day mortality was 33x higher than predicted by MELD, whilst patients with MELD scores of 20-29 had an SMR of 3.4 (95% Cl: 0-7.0), suggesting participants’ mortality risk was 3.4x higher.

**Table 2:** Standardised 90-day mortality ratios for each MELD-Na score tertile *number of patients in each MELD-Na score tertile. †Predicted number of deaths based on 90-day mortality rates for given MELD-Na scores reported by Kim et al [33]. ‡ SMR = Observed number of deaths/number of deaths predicted by MELD-Na MELD-Na: model for end-stage liver disease sodium; SMR: standardised mortality ratio; Cl: confidence interval.

		90-day mortality (number of deaths)		
MELD-Na	n*	Observed	Predicted^†^	SMR^‡^ (95% Cl)
<17	4	2	0.08	25 (0-59.6)
17-26	9	4	0.77	5.2 (0.1-10.3)
≥27	2	2	0.73	2.7 (0.1-8.1)

For all MELD deciles analysed the observed number of deaths was not significant compared to the number predicted by MELD. As no patients had a MELD score greater than 29, the calibration of MELD in patients with SBP and MELD scores of 30-39 or ≥40 was not evaluated.

For patients of all MELD-Na tertiles (<17, 17-26 and ≥27), the number of deaths observed was higher than the number predicted (Table 3). As with MELD, the magnitude of underestimation was highest in patients with the lowest scores; the SMR for patients with a MELD-Na score <17 was 25.0 (95% Cl: 0-59.6) whilst for patients with scores ≥27 the SMR was 2.7 (95% Cl: 0.1-8.1). For all tertiles, the difference in the number of deaths observed compared to the number predicted was not significant.

**Table 3:** Standardised 90-day mortality ratios for each MELD score decile *number of patients in each MELD score decile. †Predicted number of deaths based on 90-day mortality rates for each MELD decile reported by Wiesner et al [15]. ‡ SMR = Observed number of deaths/number of deaths predicted by MELD MELD: model for end-stage liver disease; SMR: standardised mortality ratio; Cl: confidence interval.

		90-day mortality (number of deaths)		
MELD	n*	Observed	Predicted^†^	SMR(95% Cl)^‡^
≤9	3	2	0.06	33.3 (0-79.5)
10-19	6	4	0.36	11.1 (0.2-22.0)
20-29	6	4	1.18	3.4 (0-7.0)
30-39	0	-	-	-
≥40	0	-	-	-

## Discussion

SBP is a common infection in patients with cirrhosis and ascites and confers high short-term mortality. At present, the accuracy of the widely used prognostic scores MELD and MELD-Na in this cohort is unclear. By evaluating these scores’ ability to predict 90-day mortality and whether the risk estimates they provide are accurate in patients with SBP, this study adds to our understanding of the areas of practice where MELD and MELD-Na may be useful tools for clinicians and researchers to utilise in this patient group and where a more cautious interpretation may be required.

In this study, MELD’s c-statistic of 0.66 fell below the generally accepted cut-off of 0.7 to consider a prognostic score clinically useful [15]. This likely reflects the findings that, of the three components of MELD, only creatinine was higher in patients who died compared to those who survived. Bilirubin and INR were almost identical. These results appear to partially conflict with previous studies, which have reported that, in addition to high creatinine at diagnosis, high bilirubin is also an independent predictor for both the development of renal failure and short-term mortality [11,39].

In another study by Nobre et al. [40], bilirubin and INR were significantly higher in patients who died during their index hospital admission compared to those who survived. Perhaps unsurprisingly, MELD also showed excellent discriminatory ability (c-statistic=0.84). These discrepancies highlight that this study’s results reflect only a limited number of patients and from only London hospitals. Therefore, they may not be representative of all patients presenting with SBP. It should be considered that if this study was repeated in a larger, more geographically diverse group of patients MELD’s predictive accuracy may be higher.

The addition of serum sodium into MELD to produce MELD-Na showed a non-significant improvement in the model’s discriminatory ability despite hyponatremia’s observed association with 90-day mortality. The lack of superiority using MELD-Na appears to conflict with previous studies where MELD-Na but not MELD was an independent predictor of short-term mortality in this cohort [21]. Previously, lower serum sodium on diagnosis has been reported as an independent predictor for the development of hepatorenal syndrome (HRS) [41], a rapidly progressive form of severe renal impairment which occurs due to renal vasoconstriction [42]. SBP is a common precipitant of HRS [43] and its development is a key predictor of short-term mortality in this patient group [44,45]. As mentioned, whilst HRS is clearly a form of renal impairment [46], it has also been demonstrated to be a severe manifestation of circulatory dysfunction [45]. As hyponatremia appears to be able to reflect this dysfunction [41] the inclusion of serum sodium may improve MELD-Na’s assessment of renal function in this patient group compared to the use of creatinine alone in MELD. Whilst we were unable to use multivariate analysis to assess whether hyponatremia remains a predictor of mortality independent of MELD, in the context of the study’s small sample size, these findings suggest that the lack of significant improvement in MELD-Na’s predictive accuracy compared to MELD may be due to the study being underpowered.

The optimum cut-off for MELD-Na (>18.5) but not for MELD (>16.5) was able to stratify patients into two groups with significantly different 90-day mortality rates. However, when considering if this stratification could be useful in clinical practice, it should be noted that the 90-day mortality of patients with MELD-Na scores below or equal to this cut-off was not inconsiderable (33.3%). Therefore, to classify this group as “low” risk and in need of less intensive monitoring or treatment appears inappropriate. In a larger study by de Oliveria Coberllini Jacques et al. [10], although the optimum MELD-Na cut-off was similar (>19), its specificity was still extremely poor (0.43), suggesting several deaths were observed in patients with scores below this threshold. Whilst future studies are required to better elucidate if MELD-Na, in particular, may have a role in the risk-stratification of this cohort, these results appear to support EASL guidelines that patients who develop SBP are not currently risk-stratified for treatment decisions; all should receive broad-spectrum antibiotics and intravenous albumin [9].

Both MELD and MELD-Na underestimated the 90-day mortality of patients in this study, although no differences in the number of observed and predicted deaths at each decile or tertile respectively reached statistical significance. As discussed previously, this result should be considered with the study’s sample size in mind, as larger studies have reported MELD to underestimate mortality in specific SBP cohorts and where patients with cirrhosis and bacterial infections more generally were included [22,47]. To our knowledge, no results from larger studies evaluating the calibration of MELD-Na in this cohort are available for comparison with the present findings. Therefore, whilst a suggestion that the study was underpowered to detect differences in observed and MELD-Na predicted mortality can be made with less confidence, it should still be considered as a strong possibility on the basis that each tertile contained at most nine patients.

From these results, we can only hypothesise why these scores do not appear to accurately represent this cohort’s poor prognosis. In univariate analysis, WBC count was almost significantly higher in non-survivors compared to survivors and in larger studies; this has consistently been reported as an independent predictor of mortality in this cohort [21,48]. Whilst not formally proven, high levels of systemic inflammation in patients with cirrhosis have been suggested as a key driver for the development of acute-on-chronic liver failure (ACLF) [49]; a syndrome characterised by the presence of extra-hepatic multi-organ failures and, importantly, a very poor short-term prognosis [50]. Previously, when the precipitants of ACLF were evaluated in a large cohort, SBP was observed as the most common infective cause [50]. The timeline of SBP and ACLF development can also occur in reverse, as shortly following diagnosis with ACLF, patients are highly predisposed to developing bacterial infections such as SBP [51]. Whilst unable to formally identify the presence of ACLF in this study cohort, patients who died were more likely to have an AKI or hepatic encephalopathy, suggesting extra-hepatic impairment, specifically, renal and cerebral dysfunction respectively. In addition, the high 90-day mortality rate of 66.7% appears similar to the poor survival that high-grade ACLF confers [38,50] and to another European study where the 90-day survival of patients with SBP and ACLF on presentation was 29% compared to 56% in patients with SBP alone [22].

Whilst inflammation and its clinical consequences appear to have an important prognostic role in this cohort, MELD and MELD-Na do not include either direct measures of inflammation in their calculation, such as WBC count or c-reactive protein, or account for the extra-hepatic dysfunction as part of ACLF, as they do not include variables which reflect the function of the many different organ systems that may be affected. In ACLF cohorts, the CLIF-Consortium ACLF (CLIF-C ACLF) prognostic score which considers a patient’s circulatory, respiratory, and cerebral function in addition to renal, hepatic and coagulation dysfunction represented by MELD showed a significantly improved ability to predict 90-day mortality [50].

Outside of the context of ACLF, systemic inflammation in SBP is associated with other clinical consequences which these scores may not account for. Higher levels of pro-inflammatory cytokines on diagnosis have been associated with increased haemodynamic dysfunction and are suggested as a key factor in the pathogenesis of HRS development [45,52]. Previously MELD has been shown to underestimate the mortality of patients with HRS [53,54], therefore the presence of this syndrome, which as discussed is common in patients with SBP, may also explain these findings.

A particularly interesting consideration is whether these models may underestimate the mortality of all patients with SBP or if only certain groups are affected. In this study, the magnitude of underestimation was non-uniform across different MELD and MELD-Na score categories, with the largest difference between actual and predicted mortality observed in patients with the lowest prognostic scores (MELD ≤9 and MELD-Na <17). Kimmann et al. [22] reported MELD to only underestimate 90-day mortality in SBP patients in the “intermediate” MELD group with scores of 15-25, whereas in a cohort of patients with cirrhosis and bacterial infections MELD underestimated the short-term mortality of all study participants irrespective of their score [47]. These differences may be due to each study using different MELD score values to split up the study cohort. In addition, differences in the patient characteristics within each score grouping may affect the presence or magnitude of underestimation. In future, the use of standardised score groupings across studies may allow it to be determined if MELD and MELD-Na could still provide accurate mortality risk estimates in some patients with SBP.

When considering this study’s findings, one should also be mindful of its limitations. Firstly, the prevalence of SBP in the time periods analysed was considerably lower than reported previously [2,3]. This may be due to the widespread use of primary prophylaxis [9], which has been shown to be effective at preventing SBP in high-risk groups [55]. The potential implications of the resultant small sample size have been discussed previously, although additionally, the finding that SBP prevalence may be very low in these London NHS trusts should be also considered in the design of future prospective studies in this patient group. For a study to contain enough participants to avoid or reduce these limitations as far as possible, patient recruitment appears to require a time period greater than three years or involve several NHS trusts.

As the electronic records used to collect information for each study participant do not include prescription charts, no information was obtained on the treatment and interventions that the study cohort received. We assumed patients were treated in line with current EASL guidelines [9], however, in practice, multiple barriers to guideline implementation exist, ranging from personal to external factors [56]. In patients with SBP, delays in diagnostic paracentesis [57] and initiation of antibiotics [58] have previously been associated with poorer short-term outcomes and therefore may have influenced our findings.

Due to the study’s retrospective nature, the laboratory tests used to calculate MELD and MELD-Na scores were dependent on when these had been undertaken during a patient’s admission. Whilst all scores were calculated using tests performed within 24 hours of diagnostic paracentesis, this could have resulted in scores used which do not in fact represent a patient’s true MELD or MELD-Na score on diagnosis with SBP.

Alongside addressing these limitations, a key consideration for future work is to evaluate and characterise any real-world consequences that may affect this patient group due to potential inaccuracies in these scores. Patients with end-stage liver disease have described confusion over their disease trajectory previously [59] although it is unclear to what extent, if at all, the use of these models may be a contributing factor. In any case, by identifying the causes of patients’ uncertainty, strategies to improve this are likely to be more effective and may aid in improving patient’s willingness to engage with palliative care services for example [29].

Another aspect to consider is whether patients with SBP are disadvantaged in referral to liver transplant assessment units or in the allocation of donor livers. As a UK-based study, we did not assess this as UKELD [19] is utilised for this purpose rather than MELD or MELD-Na. However, as UKELD incorporates the same four variables as MELD-Na, future studies could investigate the mortality of patients with SBP who do not reach the cut-off required for referral for liver-transplant eligibility assessment (UKELD <49) [60]. In addition, after adjusting for confounding factors such as the size of the liver-transplant waiting list and recipient blood group [60], wait-list mortality of patients after an episode of SBP could be compared to patients with no history of SBP to determine if patients may be disadvantaged in the prioritisation of donor-livers.

Given its apparent prognostic role in this cohort, the predictive accuracy and calibration of models which include measures of systemic inflammation should be evaluated as potential alternatives. One such example is the CLIF-Consortium Acute Decompensation score (CLIF-C ADs) which incorporates WBC count (alongside age, bilirubin, creatinine and INR) [61]. In the derivation, internal and external validation cohorts, CLIF-C ADs have shown a superior ability to predict 90-day mortality when compared to MELD and MELD-Na in patients hospitalised with decompensated cirrhosis [61]. In a separate cohort, the predicted mortality rates for each CLIF-C ADs matched closely to the actual mortality observed in the study [62]. Future work could continue to validate this score in carefully defined SBP cohorts to determine if these results are also found when this patient group is considered more specifically.

## Conclusions

In conclusion, in this small cohort of patients with cirrhosis and SBP, MELD had limited ability to predict 90-day mortality. Whilst hyponatremia was associated with mortality, the inclusion of serum sodium concentration in MELD-Na showed a non-significant improvement in predictive ability compared to MELD alone. The risk estimates provided by both models appeared to underestimate study participants’ 90-day mortality, therefore, alongside validating the present findings, future studies should consider evaluating the calibration of alternative prognostic scores in this patient group.
